# Who are the under- and never- screened for cancer in Ontario: a qualitative investigation

**DOI:** 10.1186/1471-2458-14-495

**Published:** 2014-05-23

**Authors:** Dionne Gesink, Alanna Mihic, Joan Antal, Brooke Filsinger, C Sarai Racey, Daniel Felipe Perez, Todd Norwood, Farah Ahmad, Nancy Kreiger, Paul Ritvo

**Affiliations:** 1 Dalla Lana School of Public Health, University of Toronto, 155 College St, Toronto, ON M5T 3M7, Canada; 2 Cancer Care Ontario, Toronto, ON, Canada; 3 York University, Toronto, ON, Canada

**Keywords:** Cancer screening, Qualitative methods, Sexual abuse, Mennonites, Ethics

## Abstract

**Background:**

Observed breast, cervical and colon cancer screening rates are below provincial targets for the province of Ontario, Canada. The populations who are under- or never-screened for these cancers have not been described at the Ontario provincial level. Our objective was to use qualitative methods of inquiry to explore who are the never- or under-screened populations of Ontario.

**Methods:**

Qualitative data were collected from two rounds of focus group discussions conducted in four communities selected using maps of screening rates by dissemination area. The communities selected were archetypical of the Ontario context: urban, suburban, small city and rural. The first phase of focus groups was with health service providers. The second phase of focus groups was with community members from the under- and never- screened population. Guided by a grounded theory methodology, data were collected and analyzed simultaneously to enable the core and related concepts about the under- and never-screened to emerge.

**Results:**

The core concept that emerged from the data is that the under- and never-screened populations of Ontario are characterized by diversity. Group level characteristics of the under- and never- screened included: 1) the uninsured (e.g., Old Order Mennonites and illegal immigrants); 2) sexual abuse survivors; 3) people in crisis; 4) immigrants; 5) men; and 6) individuals accessing traditional, alternative and complementary medicine for health and wellness. Under- and never-screened could have one or multiple group characteristics.

**Conclusion:**

The under- and never-screened in Ontario comprise a diversity of groups. Heterogeneity within and intersectionality among under- and never-screened groups adds complexity to cancer screening participation and program planning.

## Background

Cancer screening rates are below targeted rates for breast, cervical and colon cancer for the province of Ontario in Canada [1], despite the cost of screening being covered by health insurance (Appendix I). We believe the difference between observed and targeted cancer screening rates is attributable to two populations: an under-screened population, comprising individuals who are eligible for screening, and have been screened in the past, but are not up to date on their screening currently (e.g. women over 21 years of age with more than three years between cervical cancer screening tests); and a never-screened population comprising individuals who have never been screened for cancer despite being eligible (e.g. men over 50 years of age who have never been screened with a fecal occult blood test or colonoscopy).

Most screening studies intended to increase cancer screening rates focus on specific vulnerable and marginalized populations known to have low cancer screening rates [2-10], such as: immigrants [11-17], ethnic minorities [2,11,15,18,19], underserved populations [20,21], uninsured [11,18,20], individuals with mental health issues [22-24], indigenous populations [4,15,25,26] and rural residents [15,27,28]. These vulnerable and marginalized populations tend to be localized geographically, resulting in community or neighbourhood level studies designed to inform changes at the local level and to be generalizable to other vulnerable and marginalized populations in other geographic locations. Few, if any, studies have sought to explore the under- and never-screened at larger geopolitical and population levels, such as the state or provincial level, or confirm that vulnerable and marginalized groups have low cancer screening rates when observed over a larger area.

Our objective was to explore who the perceived under- and never- screened populations are for Ontario, the second largest province in Canada (covering 10% of Canada’s land mass (total area = 1,076,395 km^2^) and capturing two time zones). Our intention is to use the results to inform cancer screening programs, intervention activities, policy and practice at the provincial level, and begin to validate the generalizability of other studies findings to the Ontario context.

## Methods

Grounded theory methodology [29] was used to conduct this qualitative inquiry in an effort to build a substantive theory of who are the under- and never-screened populations of Ontario. A constant comparison method was employed iteratively moving back and forth between data collection, analysis, and selective theoretical sampling of participants and literature until the core concepts defining the under- and never-screened populations of Ontario emerged from the data [29]. Focus groups were conducted until theoretical saturation was reached [29,30].

We identified communities with low cancer screening rates for all three cancers (below 50% for breast and cervical cancer and below 10% for colon cancer) using maps [31] of breast, cervical, and colorectal cancer screening rates at the dissemination area level for the province of Ontario (maps not shown). Twelve communities were identified. We excluded communities with immediately apparent explanations for low rates (e.g. industrial areas or university campus). We also excluded areas with regional cancer screening projects already focused on increasing cancer screening rates among the under- and never-screened in order to avoid conflicting studies. The remaining four communities were treated as archetype communities, generally representative of other similar communities across Ontario: one urban community in a mega-city (population 2.6 million in 2011), one suburban community (population slightly over 700,000 in 2011), one small city (population slightly over 100,000 in 2011), and one rural community (population slightly over 4,000 in 2011).

We learned who the perceived under-screened and never-screened populations were for each area using an iterative qualitative process involving two rounds of focus group discussions [32,33]. The first round of discussions was with health service providers from each of the four low screening rate communities. Health service providers included professionals providing frontline health care or assistance, predominantly in clinic settings, such as: physicians, clinic nurses, nurse practitioners, public health nurses, health promoters, health educators, outreach workers, clinic directors, and social workers. Health service providers were recruited in each community first by interviewing the Health Director of the local Community Health Clinic. Knowledge was exchanged between the Health Director, who acted as a key informant, and the research team about cancer screening, identifying under- and never- screened populations, and cancer screening priorities for both the clinic and research projects. Each Health Director shared their insights, experiences and networks of relevant health professional contacts, which resulted in a diverse pool of potential health service provider participants. Each Health Director then extended an invitation to participate in a focus group to the professionals identified.

The second round of focus group discussions was with community members from each of the under- and never- screened populations identified by health service providers in each of four low screening rate communities.

Recruitment of community members varied by community, because each under- or never- screened population identified had its own socio-cultural norms and processes around connecting with research, modes of communication, and methods of participant recruitment. We relied on local health service providers and key community informants to guide us through the appropriate process. For example, for the urban and suburban communities, community outreach workers who participated in the health service providers focus group recruited community members using a relationship based approach [34] and co-facilitated focus groups in the language of their community (e.g. Spanish, Urdu, and Hindi). For the small city, we partnered and coordinated with the Salvation Army who recruited a volunteer sample of participants from their Breakfast Program using a venue-based approach [35,36]. Finally, we recruited a volunteer sample of participants from the rural community using invitations to health care clinic clients and flyers posted in the local health clinic and around the community. Potential participants called the clinic to register for the focus group.

The University of Toronto research ethics board reviewed and approved this project. The purpose of the study and focus group discussion was reviewed with participants, as was confidentiality of the discussion. Potential participants were invited to ask questions and verbal consent to participate was sought.

Cancer screening rates and maps were shared with consenting participants, followed by a group discussion about who participants believed were the under- and never- screened, and the perceived barriers and facilitators to screening. Barriers and facilitators to cancer screening will be presented in depth elsewhere. The focus group discussion guide was the same for health care providers and community members, with the language around questions modified slightly to fit the context of the group. For example, health care providers were asked about who they do not see coming in for cancer screening, while community members were asked who they thought did not seek cancer screening. Different team members led different focus groups (as appropriate, for example, male researcher led male focus groups) but all were trained in the spirit of what information was being sought from the discussions so there was consistency across groups. Focus group discussions lasted approximately one and a half to two hours each and were voice recorded and transcribed. Field notes were also taken during each discussion and used to provide context, inform the analysis, and assist interpretations of results. Focus group discussions were held between June 2011 and May 2012.

A classic grounded theory approach was taken in the analysis of the data, where transcripts were read and re-read for familiarity and open coded [37,38] by six independent analysts [39] using memoing to document the analysts’ conceptual and theoretical ideas that emerged throughout the process [40]. Analysts came together after independent open coding to review and reconcile similarities and differences in emergent categories and their properties. Together they developed a delimited coding approach and returned to the data to begin selective coding [40]. The analysts work was brought together by the main author and analyst DG to develop the substantive theory of who are the under- and never-screened in Ontario. We adhered to the relevance, appropriateness, transparency and soundness (RATS) guidelines while conducting and presenting this research (Appendix II).

## Results

We conducted four focus groups with health service providers and 16 focus groups with community members from communities with low screening rates for all three cancers (Table 1). The majority of community participants were under- or never-screened. All urban and suburban community participants were immigrants. The majority of small city and rural community participants were born in Canada and white. Saturation was reached by the third focus group with health service providers and by the 12^th^ focus group with community members. We continued to 16 focus groups both to confirm saturation and to honour the under- and never-screened community groups that had already been organized to share their perceptions and recommendations.

**Table 1:** Summary characteristics of focus group attendees

**Focus group**	**Location**	**Culture**	**Age**	**Gender**	**Language**	**Number of participants**
Providers	Urban	Multicultural	25+	Both	English	5
	Suburban	East Indian and White	20+	Female	English	4
	Small City	White	30+	Female	English	4
	Rural	White	30+	Female	English	6
Community	Urban	Latina	30+	Female	Spanish	15
		Hindi-Urdu	50+	Female	Hindi-Urdu	16
		Indo-Caribbean	50+	Female	English	14
		Afro-Caribbean	50+	Male	English	9
	Suburban	Indo-Caribbean	50+	Male	English	6
	Small City	Street Involved	30+	Female	English	17
			(2 groups)			
		Street Involved	50+	Male	English	10
			(2 groups)			
	Rural	Rural (White)	<40	Female	English	26
			(2 groups)			
			40+			
			(3 groups)			
		Rural (White)	40+	Men	English	8
			(2 groups)			

The core concept that emerged from the data is that the under- and never-screened of Ontario are a diverse body of people, similar to the diversity present in the general population of Ontario. The related concepts that emerged delineate the qualities of the under- and never-screened populations, including marginal or vulnerable groups, most notably: the uninsured; sexual abuse survivors; and people in crisis; and large segments of the general population, including: immigrants; men; and individuals accessing traditional, alternative and complementary medicine for health and wellness. The related concepts are described with supporting quotes and the core concept is then further discussed in the context of the relevant literature.

### The uninsured

Ontario residents have access to universal health care through the Ontario Health Insurance Program (OHIP). Unexpectedly, health service providers in urban, suburban and rural areas identified the uninsured, or individuals who do not access OHIP coverage, as under- or never- screened in their community:

“*Yes, it has been a taboo issue, even among service providers who acknowledge that part of their clients are uninsured.” (Urban health service provider)*

In the urban setting, the uninsured are often immigrants who had a claim for refugee status denied but never left Canada. Community members confirmed that some people were in Canada illegally and therefore not insured, and consequently under- or never- screened for cancer. They indicated that the cost of health care and cancer treatment was so high for this group, that they could not afford to be screened:

“if the person isn’t legal here, even the eye on the face costs them… if one is not here legally, you have to pay for everything that presents itself.” (Urban community member)

In the rural setting, health service providers identified Old Order Mennonites as uninsured:

“…we have two to three thousand people who are Mennonite… they pay taxes… they don’t have OHIP.” (Rural health service provider)

Rural community members reviewing maps of cancer screening rates confirmed:

“That is the Mennonite population and they pay cash when they go to the doctor and the hospital.” (Rural community member)

Rural community members further explained that seeking health care for health problems was rare for Old Order Amish and Old Order Mennonites:

“There’s a lot of Mennonites in this area and I’m not sure that isn’t affecting your [map] …The Mennonites are more forward but even them, I’m not sure. But I’m sure the Amish wouldn’t see you unless they were dying. They just don’t do that.” (Rural community member)

Old Order Amish and Old Order Mennonites are sometimes called horse and buggy people, or plain people. Traveling to town by horse and buggy for preventative care was unlikely:

“It’s a half hour drive - if you have a car or somebody can take you there. What about people who don’t have a car or transportation? What about Mennonite women? People who don’t have insurance? Those are barriers.” (Rural community member)

### Sexual abuse survivors

Sexual abuse survivors were identified as an under- or never- screened group, which makes sense since breast, cervical, and colon cancer screening involve our most intimate sexual body sites. As one health service provider explained:

“I’ve been a psychotherapist for 17 years in the community and a large group of female clients who were recovering from childhood sexual abuse and sexual trauma, would never go for a pap test. And part of my working in several cases was to help them consider the possibility of doing it. Even women in their fifties and sixties who never had that test done….if they’re hurt may be one thing, but to be screened. No.” (Urban health care provider)

Many sexual abuse survivors do not want physical contact with those areas of their body where screening is focused. Some sexual abuse survivors have psychologically dissociated with these areas as a way to cope with their trauma:

“And there is a phenomenon that goes along with sexual abuse, particularly with childhood sexual abuse where you psychologically … remove those body parts from your body. Like they don’t exist and you ignore that they are there because they’re the source of your trauma and it’s a division. Like I have a body, it does not include those parts.” (Rural health care provider)

### People in crisis

Health service providers also observed that people in crisis are usually focused on meeting their basic needs of food, shelter, and safety, and therefore, are unlikely to be screened for cancer:

“The folks at our centre, they’re mostly in crisis. So, they’re leading … marginalized lives, so cancer screening is really at the bottom of the list. That’s because there’s serious mental health stuff going on; there’s absolutely no money; there’s no food. How do you think about going for a Pap when you can’t feed your kids, for example?” (Suburban health service provider)

This observation was later validated by several women in a small city:

Respondent 1: Bus transportation is expensive. You, for a bus pass if you’re not on disability,

Respondent 2: You can’t afford it.

Respondent 3: You’re taking that from somewhere needed.

And most succinctly put by one single mother in a small city when she stated:

“I can’t afford to fall down and go boom”

The implications of a positive screening test can be a barrier to screening for people living in crisis or poverty.

### Immigrants

As expected, health service providers identified several immigrant groups as having low cancer screening rates:

“…among the Indian population, cervical cancer rate is usually very high for a number of reasons. And they’re the ones who are least screened.” (Suburban health care provider)

Immigrant community members explained that the health paradigms, attitudes, and health care seeking practices that existed in their country of origin, coupled with a lack of knowledge of the Canadian health system or the Ontario Health Insurance Program (OHIP) contributed to under-screening, or not getting screened at all:

“*…as a newcomer when you come, you don’t know what your OHIP covers. You don’t know you get a full body check and even so issues like breast cancer, cervical cancer, it’s not something that people like the government in [many] countries would talk about…” (Small City community member)*

“…if you look at the male West Indian, particularly, or the male Asian particularly, or the Afro man, in developing countries, you know, you are taught to do certain things and so when you look at a mature adult, who has come to this country, you know, that mentality carries on.” (Suburban community member)

Stigma and cultural taboos were also identified as important factors that kept immigrants, particularly men, from seeking screening:

Respondent 1: “The stigma that is attached to, you know, going to the doctor’s office for things that are considered to be taboo from a testing perspective…”

Respondent 2: “Especially for a male, yeah.”

Respondent 1: “We don’t want to deal with it…even if that word gets out on the street, well, there are so many different ways you’re going to get rubbed about this. It doesn’t sit very well with the ethnic community, in a developing country.” (Suburban community members)

Immigrant community members also explained that literacy and other communication barriers, including English as a second language, prevented some immigrants from learning about screening:

“The reminder comes in English, and many people cannot read English. What do they do? Their husband or children look at the envelope, see it’s from some hospital and put it aside. They don’t even bother to open it and see what it is about, let us know what its saying, and tell us to go.” (Urban community member)

### Men

Both health service providers and community members identified men as an under-screened population:

“You have to threaten the men to the point where you push them in the door, right?” (Rural community member)

“Most men around here are afraid to go to doctors, you know. Unless somebody’s dying you know. Actually dying. And if they don’t have somebody to push him to get him to the doctor, maybe he’ll die. They don’t like to go to the doctor .” (Urban community member)

Though many men, if not most men, limit their interaction with the medical system, there is some contact. Rural health service providers described using these moments of contact to address multiple health checks:

“See that’s the problem, the men don’t tend to come as much for their health care, it’s hard to catch them for those things. We try and catch them at any visit that they come for…” (Rural health service provider)

Rural health service providers also described the conditions under which men will come in to the clinic to seek health care:

Interviewer: “And when do they finally come in?”

Respondent 1: “When they can’t actually walk anymore.”

Respondent 2: “Their wife called and made the appointment cause she couldn’t stand listening to them anymore.”

Respondent 1: “Or they’re bleeding from an orifice somewhere.”

Respondent 3: “Or if it’s impacting their work.”

Respondent 4: “After a long time. They’ll put up with a lot before they’ll finally breakdown.”

Men had a generalized reputation of not accessing health care, and therefore, being under-screened or never- screened for cancer. Women were perceived as having more, even regular contact with the medical system and hence, were perceived as being up-to-date with screening. However, similar to Grunfeld’s finding with cancer survivors [41], we found evidence that many women were only up-to-date for one or two of the three cancer screening tests, despite being eligible (fieldnotes).

Additionally, suburban and rural men indicated that we would find a difference in health care seeking behaviours between working men and retired men, with retired men being more likely to interact regularly with the medical system, including for screening.

### People accessing traditional, complementary and alternative medicine

Health service providers indicated that many individuals and groups living in Ontario seek traditional and alternative forms of healthcare before, instead of, or in addition to seeking health care services from mainstream contemporary health care providers and systems:

“But I also think it’s not only the uninsured that subscribe to the use of alternative medicine. It’s others. I mean we have so many cultural groups. I know of many as well, you know the African community, the Caribbean community who, they believe in traditional medicine, you know not the mainstream medicine, and they will be diagnosed and they will choose not to…they will go back home to drink something, or eat something, but that’s their belief and we have to respect that.” (Urban health service provider)

Urban, suburban and rural community members indicated that many community members, especially men, do not want to seek medical care from a physician, preferring to rely on themselves, and described the use of traditional and alternative medicine to facilitate self-healing:

“…one of my best friends lost his life this very last year, we went to the funeral. He had prostate cancer for years and years and not even his wife know, and he hide it from the doctor. And when he buy all this herb they tell him about and he boil it and he hide it from his wife. When he died his wife found a whole thing of herb. A whole thing of herb, he’s been drinking herbs for years and years and don’t check on his health, he’s scared of doctor.” (Urban community member)

“We have a majority of farmers here and the farmers take care of themselves. I’ve seen it where they’ll inject themselves with whatever they give their cows and stuff like that. … Antibiotics and stuff. … No seriously. And they will not go to the doctors. Their philosophy is … Self-healing” (Rural community member)

## Discussion

The populations perceived by health service providers and community members to have low cancer screening participation in Ontario were similar to other studies and included: the uninsured [11,18,20], sexual abuse survivors [42-47], people living in crisis, immigrants [11-17], and men [5]. There was substantial screening heterogeneity within each of these groups, from never-screened to under-screened for all eligible cancers; to up-to-date on some cancer screens but not all eligible screens; to fully screened for all eligible cancers. There can also be significant overlap between groups.

Traditional, complementary and alternative medicine (TCAM) users were perceived as being less likely to participate in breast, cervical or colon cancer screening programs. This group has not been identified previously. In 2006, 74% of Canadians surveyed had used alternative therapies in their lifetime and 54% had used alternative therapies in the past year [48]. This group is very diverse, comprising First Nations, multi-generational Canadians, farmers and other rural residents, specific ethnic and cultural groups, spiritual groups, philosophical groups, recent immigrants, established immigrants, and others. TCAM users may be less likely to access mainstream cancer screening programs; however, typically this group is conscious of, and active in, their own health, wellbeing and healing, which extends to disease prevention. Many will focus on cancer prevention and treatment through diet; exercise; balancing mental, emotional, physical and spiritual health; and following teachings for living a good life [49-52]. They may also seek alternative methods of cancer screening and treatment, and thus be up to date on their cancer screening.

The uninsured are a well-recognized group of under/never screeners in the United States [53-55]. However, we did not expect to learn of an uninsured population in Canada, which has a national ‘universal’ health care system. All Canadian residents have health insurance and access to health care. In particular, Old Order Mennonites presented an unexpected case of “uninsured” because they pay taxes and are eligible for OHIP coverage. As explained by an Old Order Mennonite Community Leader (personal communication, Stratford, Ontario, October 24, 2012), the Old Order Mennonite community does not use OHIP because of its effect on their Brotherhood, which refers to the community collective, or church, and the mechanism by which community members ask, receive and provide help for each other. The Brotherhood is vital to the survival of the community, and so, the Old Order Mennonites give to the Brotherhood and turn to the Brotherhood in times of need. Turning to insurance in times of need undermines, and so jeopardizes, the Brotherhood. Consequently, the Old Order Mennonites do not use any form of insurance and pay for health care “out of pocket”.

In Ontario, the uninsured population is relatively small, making cancer screening program decisions around prioritizing cancer screening efforts and resources to the uninsured challenging. There are also ethical considerations to balance regarding the cost of out-of-pocket cancer screening and the frequent refusal of cases to follow-up with cancer treatment. Additionally, cervical cancer risk appears to be very low to negligible for the Old Order Mennonites whose religious and cultural practices are highly protective when it comes to sexually transmitted diseases.

The sexual abuse survivor population is large [56] with estimates as high as one in three girls and one in six boys experiencing sexual abuse [57]. The trauma of abuse impacts health care seeking behaviours among survivors, especially with respect to preventative care [46,58]. Dissociation is one complex psychological mechanism many sexual abuse survivors use to cope with their abuse [43,46,59]. We were told dissociation may be a barrier to screening because survivors dissociate the parts of their body involved in cancer screening so screening is not even considered because those parts ‘do not exist’. However, dissociation may also facilitate cancer screening by helping some survivors endure an examination if they make it to a screening appointment [43]. Unfortunately, some survivors may subsequently dissociate in a negative way, if the screening exam causes them to relive some aspect of their trauma [43,46].

So far, we have presented the under- and never-screened as discrete groups. However, the under- and never-screened can also exist at the intersection of any of these group combinations; for example, abuse survivors living in crisis, or immigrant men relying on folk medicine for health and wellness. Empirical data may be used to identify the individual groups; however, it is unlikely that cancer screening programs will routinely collect data that enable the overlap to be identified. Intersectionality has its roots at the experiential intersection of gender and race [60,61] and, with minor modification can be expanded to include other social, cultural, demographic and economic factors. Modified intersectionality may provide an important and useful framework or tool by which to think through the identity and intervention or program needs of the under- and never-screened.

We do not know which participants were under- or never-screened for breast, cervical or colon cancer. We only know that we specified under- and never-screened during recruitment. Participants did not differentiate between under- and never-screened characteristics, barriers, or facilitators during group or individual discussions, suggesting there may not be a difference between these groups beyond frequency of screening.

We also do not know what proportion of the Ontario population the different under- or never-screened groups represent, with the exception of “men”. Although we know the population of men in Ontario, many men are screened and do get screened regularly for colon cancer, so the characteristics of men who are under- or never-screened is still unknown.

We did reach saturation with the groups we talked to about who is under and never screened for cancer. However, our initial seed groups were health care providers and so all focus groups were connected with mainstream western medicine, in some way. Thus the communities identified will have a history of interaction with mainstream western medicine and so be more likely to be screened for cancer. It is possible that we missed those groups who have limited or no contact with mainstream western medicine. For instance, indigenous communities were not mentioned even though indigenous people are known to have lower cancer screening rates than the general population [62]. Additionally, more groups started to emerge as our project became more widely known, including indigenous communities.

One of the lessons learned conducting these focus groups is that many under- and never-screened groups are hard-to-reach. It takes time to identify and reach those communities, and then to connect and build relationships with them. Once a relationship has been established and the community is ready to collaborate, the collaboration must be followed through with because collaborations themselves are an intervention bringing under- and never-screened to a forum where ideas and concerns can be heard, misinformation corrected, knowledge exchanged and mutual understanding developed. Stopping collaboration early can trivialize the relationship, and therefore the group, potentially causing irreparable damage and further marginalizing a population that is already hard to reach.

One question emerging from our research is: which, if any, of the under- or never- screened are open to, or willing to consider, regular screening, and which are not? It may be easier to return the under-screened to regular screening than the never-screened because the under-screened have participated in screening in the past. However, it is also possible that the under-screened had experiences, or made decisions, that will stop them from ever getting screened again. Along these lines, understanding the reasons for stopping or delaying screening for the under-screened will lend insight into how to prevent individuals from stopping screening in the first place. This will also help distinguish between under-screened individuals who have made a vow, or permanent commitment, not to get screened in the future, compared to under-screened individuals who are still of two-minds about screening.

It is also likely that there are never-screened populations who will never be screened for cancer, no matter how much effort is expended. If this is true, a public health ethics question emerges around cancer screening and informed freedom of choice, with respect to what might constitute too much external pressure to comply, or unfair manipulation or persuasion. For these individuals, it may be more important to maintain open communication without an emphasis on change.

## Conclusion

Prioritizing cancer prevention activities is difficult for cancer screening programs and the under- or never-screened groups identified. Add the complexity of being at the intersection of two or more under- or never-screened groups and cancer screening (and survival) quickly becomes for “one of the lucky ones” (addict, living in crisis, with liver failure) for the under- and never-screened.

## Appendix I: Breast, cervical and colon cancer screening program summary for Ontario, Canada

### Breast cancer screening

Women 50 years old and older are eligible for breast cancer screening with the Ontario Breast Screening Program (OBSP) and may refer themselves to the OBSP for a mammography. Women between the ages of 30 to 49 who are at high risk for breast cancer are also eligible but need a referral from their doctor or nurse practitioner to be screened through the OBSP. Women are recommended to have a mammogram every 2-3 years, however, yearly mammograms are recommended for women at high risk for breast cancer.

### Cervical cancer screening

Women 21 year old and older, who have been sexually active, are eligible for cervical cancer screening every three years, unless their health care provider recommends more frequent screening. Women must make an appointment with their clinician for pap testing, which is the only cervical cancer screening test covered by health insurance currently.

### Colon cancer screening

Women and men 50 years old and older, and individuals at increased risk for colon cancer (i.e. with a family member – parent, child or sibling – with colorectal cancer) are eligible for colon cancer screening. Colon cancer is screened using the fecal occult blood test, which looks for blood in stool, as the first pre-screening test. Colonoscopy, which looks for cancer, is recommended for people with unexplained blood in their stool or at increased risk for colon cancer because of family history. Some doctors prefer to send their patients straight to colonoscopy. The fecal occult blood test (recommended every two years) can be provided by doctors, nurse practitioners, or Telehealth Ontario. Colonoscopy (recommended every 10 years) is only available through a physician.

## Appendix II: Relevance, Appropriateness, Transparency and Soundness (RATS) Guidelines Checklist

Relevance of study question:

1. Research Question explicitly stated – yes

2. Research question justified and linked to existing knowledge base - yes

Appropriateness of qualitative method

1. Study design described and justified – yes (focus groups for group perceptions, dynamics, higher level (e.g. group) experience and observation, non-sensitive topic)

Transparency of procedures

1. Criteria for selecting study sample justified and explained – yes, purposive for diversity of opinion, volunteer because hard to reach

2. Details of how recruitment was conducted and by whom - yes

3. Details of who chose not to participate and why – not available

4. Methods outlined and examples given (interview questions) - yes

5. Study group and setting clearly described - yes

6. End of data collection justified and described - yes

7. Role of Researcher- influence on formulation of research question, data collection, interpretation?

8. Informed consent process explicitly and clearly detailed - yes

9. Anonymity and confidentiality discussed - yes

10. Ethics approval cited - yes

Soundness of interpretive approach

1. Analytic approach described in depth and justified - yes

2. Indicators of quality: description of how themes derived from data (inductive or deductive)

3. Evidence of alternative explanations sought

4. Analysis and presentation of negative or deviant cases - yes

5. Description of the basis on which quotes chosen - yes

6. Semi-quantification when appropriate - yes

7. Illumination of context and/or meaning, richly detailed - yes

8. Method of reliability check described and justified - yes

a. Six independent analysts reviewed data and contest themes; and

a. Resolution of disagreements

9. Findings presented with reference to existing theoretical and empirical literature and how they contribute -yes

10. Strengths and limitations explicitly described and discussed - yes

11. Evidence of following guidelines - yes

12. Detail of methods or additional quotes contained in appendix

13. Written for health sciences audience - yes

14. Grounded theory – not a simple content analysis but complex sociological theory generating approach.

## Abbreviations

OHIP: Ontario Health Insurance Program.

## Competing interests

The authors declare that they have no competing interests.

## Authors’ contributions

DG, AM, SR, BF, JA, PR and DFP have made substantial contributions to conception and design, or acquisition of data, or analysis and interpretation of data; TN, FA and NK made substantial contributions to acquisition of data. DG drafted the manuscript. All authors have been involved in revising the manuscript critically for important intellectual content, given final approval of the version to be published, and agree to be accountable for all aspects of the work in ensuring that questions related to the accuracy or integrity of any part of the work are appropriately investigated and resolved. All authors read and approved the final manuscript.

## Authors’ information

DG is an Associate Professor with the Dalla Lana School of Public Health at the University of Toronto; AM and SR are doctoral candidates with the Dalla Lana School of Public Health at the University of Toronto; BF and JA are Research Associates with Cancer Care Ontario; PR and NK are Senior Scientists with Cancer Care Ontario; PR and FA are Associate Professors at York University; and DFP is a doctoral student at York University.

## Pre-publication history

The pre-publication history for this paper can be accessed here:

http://www.biomedcentral.com/1471-2458/14/495/prepub

## References

[B1] Cancer Screening – Breast, Cervical and Colorectal [http://www.csqi.on.ca/Comparisons/cancer_screening]

[B2] Amankwah E Ngwakongnwi E Quan H Why many visible minority women in Canada do not participate in cervical cancer screening Ethn Health 2009 14 4 337 349 10.1080/13557850802699122 19280443

[B3] Ferroni E Camilloni L Jimenez B Furnari G Borgia P Guasticchi G Giorgi Rossi P Methods to increase participation Working Group How to increase uptake in oncologic screening: a systematic review of studies comparing population-based screening programs and spontaneous access Prev Med 2012 55 6 587 596 10.1016/j.ypmed.2012.10.007 23064024

[B4] Maar M Burchell A Little J Ogilvie G Severini A Yang JM Zehbe I A qualitative study of provider perspectives of structural barriers to cervical cancer screening among first nations women Womens Health Issues 2013 23 5 e319 e325 10.1016/j.whi.2013.06.005 23993479 PMC4111655

[B5] Red SN Kassan EC Williams RM Penek S Lynch J Ahaghotu C Taylor KL Underuse of colorectal cancer screening among men screened for prostate cancer: a teachable moment? Cancer 2010 116 20 4703 4710 10.1002/cncr.25229 20578178 PMC3639486

[B6] Sabatino SA Lawrence B Elder R Mercer SL Wilson KM DeVinney B Melillo S Carvalho M Taplin S Bastani R Rimer BK Vernon SW Melvin CL Taylor V Fernandez M Glanz K Community Preventive Services Task Force Effectiveness of interventions to increase screening for breast, cervical, and colorectal cancers: nine updated systematic reviews for the guide to community preventive services Am J Prev Med 2012 43 1 97 118 10.1016/j.amepre.2012.04.009 22704754

[B7] Subrahmanian K Petereit DG Kanekar S Burhansstipanov L Esmond S Miner R Spotted Tail C Guadagnolo BA Community-based participatory development, implementation, and evaluation of a cancer screening educational intervention among American Indians in the Northern Plains J Cancer Educ 2011 26 3 530 539 10.1007/s13187-011-0211-5 21431984 PMC3162121

[B8] Community Preventive Services Task Force Updated recommendations for client- and provider-oriented interventions to increase breast, cervical, and colorectal cancer screening Am J Prev Med 2012 43 1 92 96 22704753 10.1016/j.amepre.2012.04.008

[B9] Baron RC Melillo S Rimer BK Coates RJ Kerner J Habarta N Chattopadhyay S Sabatino SA Elder R Leeks KJ Task Force on Community Preventive Services Intervention to increase recommendation and delivery of screening for breast, cervical, and colorectal cancers by healthcare providers a systematic review of provider reminders Am J Prev Med 2010 38 1 110 117 10.1016/j.amepre.2009.09.031 20117566

[B10] Levy BT Daly JM Luxon B Merchant ML Xu Y Levitz CE Wilbur JK The “iowa get screened” colon cancer screening program J Prim Care Community Health 2010 1 1 43 49 10.1177/2150131909352191 23804068

[B11] Coronado GD Petrik A Spofford M Talbot J Do HH Sanchez J Kapka T Taylor V Perceptions of under and overutilization of cervical cancer screening services at Latino-serving community health centers J Community Health 2013 38 5 915 918 10.1007/s10900-013-9701-1 23728821

[B12] Latif E Recent immigrants and the use of cervical cancer screening test in Canada J Immigr Minor Health 2010 12 1 1 17 10.1007/s10903-009-9237-8 19259816

[B13] Lofters AK Hwang SW Moineddin R Glazier RH Cervical cancer screening among urban immigrants by region of origin: a population-based cohort study Prev Med 2010 51 6 509 516 10.1016/j.ypmed.2010.09.014 20932995

[B14] Lofters AK Moineddin R Hwang SW Glazier RH Low rates of cervical cancer screening among urban immigrants: a population-based study in Ontario, Canada Med Care 2010 48 7 611 618 10.1097/MLR.0b013e3181d6886f 20548258

[B15] Nuno T Gerald JK Harris R Martinez ME Estrada A Garcia F Comparison of breast and cervical cancer screening utilization among rural and urban Hispanic and American Indian women in the Southwestern United States Cancer Causes Control 2012 23 8 1333 1341 10.1007/s10552-012-0012-0 22710745

[B16] Sun Z Xiong H Kearney A Zhang J Liu W Huang G Wang PP Breast cancer screening among Asian immigrant women in Canada Cancer Epidemiol 2010 34 1 73 78 10.1016/j.canep.2009.12.001 20044321

[B17] Xiong H Murphy M Mathews M Gadag V Wang PP Cervical cancer screening among Asian Canadian immigrant and nonimmigrant women Am J Health Behav 2010 34 2 131 143 19814593 10.5993/ajhb.34.2.1

[B18] Chen HY Kessler CL Mori N Chauhan SP Cervical cancer screening in the United States, 1993-2010: characteristics of women who are never screened J Womens Health (Larchmt) 2012 21 11 1132 1138 10.1089/jwh.2011.3418 22873781

[B19] Ogedegbe G Cassells AN Robinson CM DuHamel K Tobin JN Sox CH Dietrich AJ Perceptions of barriers and facilitators of cancer early detection among low-income minority women in community health centers J Natl Med Assoc 2005 97 2 162 170 15712779 PMC2568778

[B20] Ackerson K Gretebeck K Factors influencing cancer screening practices of underserved women J Am Acad Nurse Pract 2007 19 11 591 601 10.1111/j.1745-7599.2007.00268.x 17970859

[B21] Tangka FK O'Hara B Gardner JG Turner J Royalty J Shaw K Sabatino S Hall IJ Coates RJ Meeting the cervical cancer screening needs of underserved women: the National Breast and Cervical Cancer Early Detection Program, 2004-2006 Cancer Causes Control 2010 21 7 1081 1090 10.1007/s10552-010-9536-3 20361353

[B22] Aggarwal A Pandurangi A Smith W Disparities in breast and cervical cancer screening in women with mental illness: a systematic literature review Am J Prev Med 2013 44 4 392 398 10.1016/j.amepre.2012.12.006 23498106

[B23] Tilbrook D Polsky J Lofters A Are women with psychosis receiving adequate cervical cancer screening? Can Fam Physician 2010 56 4 358 363 20393098 PMC2860833

[B24] Yee EF White R Lee SJ Washington DL Yano EM Murata G Handanos C Hoffman RM Mental illness: is there an association with cancer screening among women veterans? Womens Health Issues 2011 21 4 Suppl S195 S202 21724141 10.1016/j.whi.2011.04.027

[B25] McDonald JT Trenholm R Cancer-related health behaviours and health service use among Inuit and other residents of Canada's north Soc Sci Med 2010 70 9 1396 1403 10.1016/j.socscimed.2010.01.008 20172640

[B26] O'Brien BA Mill J Wilson T Cervical screening in Canadian First Nation Cree women J Transcult Nurs 2009 20 1 83 92 18669899 10.1177/1043659608322418

[B27] Carruth AK Browning S Reed DB Skarke L Sealey L The impact of farm lifestyle and health characteristics: cervical cancer screening among southern farmwomen Nurs Res 2006 55 2 121 127 10.1097/00006199-200603000-00007 16601624

[B28] Yabroff KR Lawrence WF King JC Mangan P Washington KS Yi B Kerner JF Mandelblatt JS Geographic disparities in cervical cancer mortality: what are the roles of risk factor prevalence, screening, and use of recommended treatment? J Rural Health 2005 21 2 149 157 10.1111/j.1748-0361.2005.tb00075.x 15859052

[B29] Glaser BG Strauss AL The Discovery of Grounded Theory: Strategies for Qualitiative Research 1967 Chicago: Aldine Publishing Company

[B30] Watling CJ Lingard L Grounded theory in medical education research: AMEE Guide No. 70 Med Teach 2012 34 10 850 861 10.3109/0142159X.2012.704439 22913519

[B31] Lofters AK Gozdyra P Lobb R Using geographic methods to inform cancer screening interventions for South Asians in Ontario Canada BMC Public Health 2013 13 395 10.1186/1471-2458-13-395 23622426 PMC3640962

[B32] Creswell JS Qualitative Inquiry and Research Design: Choosing Among Five Approaches 2007 2 Thousand Oaks: Sage Publications

[B33] Creswell JS Research Design: Qualitative, Quantitative and Mixed Methods Approaches 2009 3 Thousand Oaks, CA: Sage Publication

[B34] Kovach M Indigenous Methodologies: Characteristics, Conversations and Contexts 2009 Toronto: University of Toronto Press

[B35] Haley DF Golin C El-Sadr W Hughes JP Wang J Roman Isler M Mannheimer S Kuo I Lucas J Dinenno E Justman J Frew PM Emel L Rompalo A Polk S Adimora AA Rodriquez L Soto-Torres L Hodder S Venue-Based Recruitment of Women at Elevated Risk for HIV: An HIV Prevention Trials Network Study J Womens Health (Larchmt) 2014 [Epub ahead of print] 10.1089/jwh.2013.4654 PMC4046355 24742266

[B36] Muhib FB Lin LS Stueve A Miller RL Ford WL Johnson WD Smith PJ Community Intervention Trial for Youth Study Team A venue-based method for sampling hard-to-reach populations Public Health Rep 2001 116 Suppl 1 216 222 11889287 10.1093/phr/116.S1.216 PMC1913675

[B37] Glaser BG Basics of Grounded Theory Analysis: Emergence vs. Forcing 1992 Mill Valley, CA: Sociology Press

[B38] Walker D Myrick F Grounded theory: an exploration of process and procedure Qual Health Res 2006 16 4 547 559 10.1177/1049732305285972 16513996

[B39] Mays N Pope C Rigour and qualitative research BMJ 1995 311 6997 109 112 10.1136/bmj.311.6997.109 7613363 PMC2550154

[B40] Glaser BG Theoretical Sensitivity: Advances in the Methodology of Grounded Theory 1978 Mill Valley, Calif: Sociology Press

[B41] Grunfeld E Moineddin R Gunraj N Del Giudice ME Hodgson DC Kwon JS Elit L Cancer screening practices of cancer survivors: population-based, longitudinal study Can Fam Physician 2012 58 9 980 986 22972732 PMC3440276

[B42] Farley M Golding JM Minkoff JR Is a history of trauma associated with a reduced likelihood of cervical cancer screening? J Fam Pract 2002 51 10 827 831 12401150

[B43] Bates CK Carroll N Potter J The challenging pelvic examination J Gen Intern Med 2011 26 6 651 657 10.1007/s11606-010-1610-8 21225474 PMC3101979

[B44] Olesen SC Butterworth P Jacomb P Tait RJ Personal factors influence use of cervical cancer screening services: epidemiological survey and linked administrative data address the limitations of previous research BMC Health Serv Res 2012 12 34 10.1186/1472-6963-12-34 22333392 PMC3306758

[B45] Ackerson K A history of interpersonal trauma and the gynecological exam Qual Health Res 2012 22 5 679 688 10.1177/1049732311424730 22068042

[B46] Cadman L Waller J Ashdown-Barr L Szarewski A Barriers to cervical screening in women who have experienced sexual abuse: an exploratory study J Fam Plann Reprod Health Care 2012 38 4 214 220 10.1136/jfprhc-2012-100378 23027982 PMC3470431

[B47] Kelly S The effects of childhood sexual abuse on women's lives and their attitudes to cervical screening J Fam Plann Reprod Health Care 2012 38 4 212 213 10.1136/jfprhc-2012-100418 23027981

[B48] Esmail N Complementary and Alternative Medicine in Canada: Trends in Use and Public Attitudes, 1997-2006 Public Policy Sources, Volume 87 2007 Fraser Institute

[B49] Brown PJ Carney PA Health beliefs and alternative medicine: a qualitative study of breast cancer patients J Cancer Educ 1996 11 4 226 229 8989637 10.1080/08858199609528433

[B50] Wanchai A Armer JM Stewart BR Complementary and alternative medicine use among women with breast cancer: a systematic review Clin J Oncol Nurs 2010 14 4 E45 E55 10.1188/10.CJON.E45-E55 20682492

[B51] Maher P A review of ‘traditional’ aboriginal health beliefs Aust J Rural Health 1999 7 4 229 236 10.1046/j.1440-1584.1999.00264.x 10732513

[B52] Oliver SJ The role of traditional medicine practice in primary health care within Aboriginal Australia: a review of the literature J Ethnobiol Ethnomed 2013 9 46 10.1186/1746-4269-9-46 23819729 PMC3702459

[B53] Brown ME Bindman AB Lurie N Monitoring the consequences of uninsurance: a review of methodologies Med Care Res Rev 1998 55 2 177 210 10.1177/107755879805500203 9615562

[B54] Slatore CG Au DH Gould MK American Thoracic Society Disparities in Healthcare Group An official American Thoracic Society systematic review: insurance status and disparities in lung cancer practices and outcomes Am J Respir Crit Care Med 2010 182 9 1195 1205 10.1164/rccm.2009-038ST 21041563

[B55] Kennedy J Morgan S Health care access in three nations: Canada, insured America, and uninsured America Int J Health Serv 2006 36 4 697 717 10.2190/EC30-KP22-RA84-RAL4 17175842

[B56] Trocme NM MacLaurin BJ Fallon BA Daciuk JF Tourigny M Billingsley DA Canadian Incidence Study of Reported Child Abuse and Neglect: methodology Can J Public Health 2001 92 4 259 263 11962109 10.1007/BF03404956 PMC6979859

[B57] Bagdley R The Report of the Committee on Sexual Offences Against Children and Youth Two Volumes 1984 Ottawa: Supply and Services Canada

[B58] Leeners B Stiller R Block E Gorres G Imthurn B Rath W Effect of childhood sexual abuse on gynecologic care as an adult Psychosomatics 2007 48 5 385 393 10.1176/appi.psy.48.5.385 17878496

[B59] Hetzel-Riggin MD Wilber EL To dissociate or suppress? Predicting automatic vs. conscious cognitive avoidance J Trauma Dissociation 2010 11 4 444 457 10.1080/15299732.2010.495376 20938868

[B60] Crenshaw K Demarginalizing the intersection of race and sex: a black feminist critique of antidiscrimination doctrine, feminist theory, and antiracist politics University of Chicago Legal Forum 1989 139 167

[B61] Crenshaw K Mapping the margins: intersectionality, identity politics, and violence against women of color Stanford Law Rev 1991 43 6 1241 1299 10.2307/1229039

[B62] Assembly of First Nations Access to Cancer Screening and First Nations 2009

